# Tubulin Post-Translational Modifications and Microtubule Dynamics

**DOI:** 10.3390/ijms18102207

**Published:** 2017-10-21

**Authors:** Dorota Wloga, Ewa Joachimiak, Hanna Fabczak

**Affiliations:** Laboratory of Cytoskeleton and Cilia Biology, Department of Cell Biology, Nencki Institute of Experimental Biology of Polish Academy of Sciences, 3 Pasteur Str., 02-093 Warsaw, Poland; e.joachimiak@nencki.gov.pl (E.J.); h.fabczak@nencki.gov.pl (H.F.)

**Keywords:** microtubule dynamics, post-translational modifications, acetylation, glutamylation, glycylation, methylation, phosphorylation, polyamination

## Abstract

Microtubules are hollow tube-like polymeric structures composed of α,β-tubulin heterodimers. They play an important role in numerous cellular processes, including intracellular transport, cell motility and segregation of the chromosomes during cell division. Moreover, microtubule doublets or triplets form a scaffold of a cilium, centriole and basal body, respectively. To perform such diverse functions microtubules have to differ in their properties. Post-translational modifications are one of the factors that affect the properties of the tubulin polymer. Here we focus on the direct and indirect effects of post-translational modifications of tubulin on microtubule dynamics.

## 1. Introduction

Microtubules are an essential component of the cytoskeleton of a eukaryotic cell. They support key cellular functions such as maintenance of cell shape, intracellular transport, cell polarity or cell division. Although microtubules are structurally similar, they may differ in their properties, such as the rate of tubulin subunit turnover. Exposure of cells to cold [1,2] or treatment with microtubule-depolymerizing drugs [3] revealed the existence of the subpopulations of microtubules that differ in their stability. While some microtubules are very labile and disassemble within minutes under such conditions, other microtubules persist for hours or are cold-resistant. Three major factors may contribute to the heterogeneity of microtubule properties: (i) composition of the α- and β-tubulin gene-encoded isotypes that are incorporated into microtubule; (ii) post-translational modifications of tubulin that create a pattern on the microtubule surface known as the “tubulin code” [4] and (iii) interactions with diverse microtubule-interacting proteins (MIPs). It is noteworthy that these three factors can influence each other. For example, specific tubulin isotypes may differ in the extent of their post-translational modifications depending on the presence or absence of modifiable amino acids (for instance, in *Caenorhabditis elegans* only Mec-12 α-tubulin isotype has the acetylable lysine residue in position 40 [5]). In turn, post-translational modifications can affect binding/interactions of tubulin or microtubules with specific MIPs. Here we focus on the direct and indirect effects of the post-translational modifications of tubulin on microtubule dynamics.

## 2. Polyamination and Cold-Stable Microtubules

During the course of the purification of tubulin from the bovine or porcine brain by cycles of cold-induced microtubule depolymerization and warm-induced repolymerization, it was discovered that some microtubules are cold-resistant [6]. Tubulins in hyper-stable, cold-resistant microtubules, are more basic in charge compared to tubulins from cold-sensitive microtubules [6]. The basic fraction of tubulin, abundant in axons of neuronal cells, is at least in part generated by the covalent linkage of polyamine (polyamination) [7]. Polyaminated tubulin is also abundant in testes (possibly in axonemal microtubules), but only present at low levels in non-neuronal cells [7]. In vitro, both free tubulin heterodimers and tubulin heterodimers incorporated into microtubules, can be modified by transglutaminases. Moreover, polyamination of tubulin does not affect its polymerization [7].

**Figure 1:**
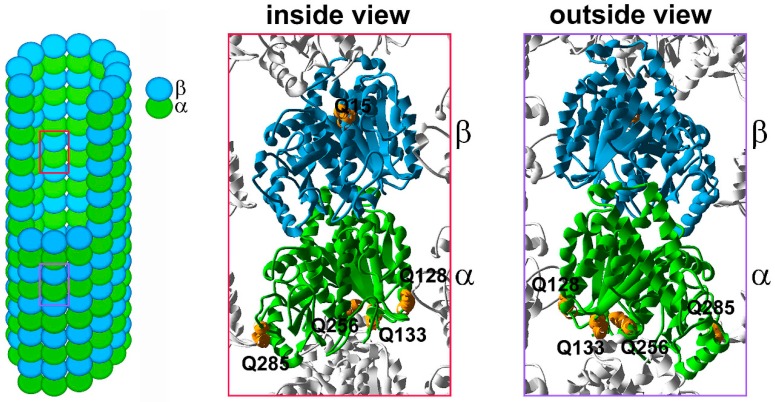
The distribution of the polyamination sites in α,β-tubulin heterodimer. Schematic representation of a microtubule, composed of α- (green) and β- (blue) tubulin heterodimers. Red and purple frames mark tubulin heterodimer with either a visible luminal side (an inside view) or outer surface (outside view), respectively. An inside and outside views of 3D model of the tubulin heterodimer within the microtubule lattice based on 3J6F.pdb (NCBI database); a tubulin molecule is composed of 12 α-helices (numbered H1–H12) and 10 β-strands (numbered S1–S10) linked by loops [8,9]. Putative tubulin polyamination sites are marked in orange. α-Tubulin Q128 is located within the H3–S4 loop and builds the H3 surface, which is involved in lateral interactions with the neighboring protofilament (an outside view, protofilament to the left). α-Tubulin Q285 is located within the S7–H9 loop, the so-called M loop, which is also responsible for lateral interactions with the neighboring protofilament (an outside view, protofilament to the right). α-Tubulin Q133, located within the H3–S4 loop and Q256 located within the H8 helix, build the minus end surface, responsible for longitudinal interactions between heterodimers within the same protofilaments or with γ-tubulin at the nucleation site. Q15 of β-tubulin is located within the H1 helix, positioned near the nucleotide binding pocket.

The highly conserved glutamine 15 (Q15) in β-tubulin, located in close proximity to the GTP pocket (Figure 1), is the main modification site but additional putative polyamination sites were mapped on both the α- and β-tubulins. Q128 and Q285 of α-tubulin are located within the H3 lateral surface and M loop, respectively (Figure 1), the dimer interfaces that face the heterodimers of the neighboring protofilaments [8,9]. The Q133 and Q256 residues are positioned near the α-tubulin minus end (Figure 1), suggesting possible involvement in the interactions between the α,β-tubulin heterodimer and γ-tubulin, or with heterodimers within the same protofilament. Thus, tubulin polyamination could play a role in the control of GTP binding or hydrolysis, and in microtubule lattice stabilization [7]. It is unknown if in vivo any microtubule interacting proteins recognize and preferentially interact with cold-stable polyaminated microtubules, and thus, further modulate their stability.

## 3. Microtubule Acetylation

Acetylation is a highly evolutionarily conserved tubulin modification. The ε-amino group of the lysine 40 residue (K40) of α-tubulin is a predominant acetylation site [10]. αTAT1 (in *C. elegans* known as Mec-17) is the main, if not the only, acetyltransferase for K40 [11,12], and the reverse reaction is catalyzed by the deacetylases: Sirt2 (Sirtuin type 2) [13] or histone deacetylase HDAC6 [14], which is the major tubulin deacetylases in vivo [15]. In the assembled microtubules that are the preferred substrate of αTAT1 [12], K40 is located within a loop protruding into the microtubule lumen [8,16]. The in vivo and ex vivo obtained data suggest that αTAT1 acetyltransferase could enter the microtubule lumen at the microtubule ends or through internal gaps in the microtubule lattice and relatively slowly diffuse inside the microtubule [11,17,18,19]. Thus, on growing microtubules, the acetylation is initiated at the microtubule ends, and the acetylated segment enlarges as the microtubule elongates and the enzyme diffuses [11,19]. It was also suggested that the microtubule acetylation rate is limited by the slow catalytic activity of αTAT1 [20].

Until recently, nearly all data concerning the role of tubulin acetylation related to the predominant acetylation of K40 of α-tubulin [11,21]. This unique luminal modification was discovered on flagellar microtubules in green algae, *Chlamydomonas reinharditii* [10,22], and was subsequently shown to be present on long-lived microtubules in nearly all eukaryotic cell types [23]. In the light of the recent data, the correlation between K40 acetylation and microtubules longevity can be explained by several factors that limit the rate of microtubule acetylation, including (i) the need to use either microtubule ends or lattice discontinuities to access the luminal space; (ii) the slow rate of αTAT1 diffusion; and (iii) the slow enzymatic rate of αTAT1. Consequently, only microtubules that last long enough can be significantly acetylated along their entire length [17,19,20].

In vivo, acetylated microtubules were shown to be more resistant to mild cold, nocodazole or colchicine treatment [24,25]. However, the analysis of microtubule dynamics in cells with changes either in the level of the modifying enzymes (αTAT1, HDAC6 and SIRT2) or enzymes activity, brought inconsistent data, both supporting and opposing the role of tubulin K40 acetylation as a stabilizing factor [15,26,27,28,29]. For example, in NIH3T3 fibroblasts, αTAT1 depletion resulted in an increased number of microtubules that were resistant to nocodazole, while in cells overexpressing either the active or inactive form of the αTAT1 acetyltransferase, microtubules were destabilized to a similar extent [30,31]. Moreover, in cells overexpressing either the active or inactive αTAT1, the microtubules grew and shrank more rapidly than microtubules in αTAT1-shRNA transfected cells [30]. However, Xu and the co-authors reported that microtubules in RPE αTAT1-depleted cells were more sensitive to nocodazole treatment while overexpression of αTAT1 (but not an inactive enzyme) resulted in increased resistance to nocodazole [32]. Accordingly, in MEFs lack of tubulin deacetylase HDAC6 increased resistance to nocodazole [27].

Interestingly, HDAC6 may act independently of its enzymatic activity. In mammalian cells, knockdown of HDAC6 did not affect microtubule growth velocity, but inhibition of HDAC6 activity by a specific inhibitor, tubacin, or expression of HDAC6 with a mutation in one out of two catalytic domains, reduced the rate of microtubule growth and shrinkage speed. Surprisingly, overexpression of HDAC6 carrying mutations at both catalytic domains, similar to overexpression of wild-type deacetyltransferase, had no effect on microtubule growth [33]. It was suggested that HDAC6 bound to the microtubule growing end may physically, independently of its activity, affect both the attachment and detachment of tubulin heterodimers [33].

It is also noteworthy that tubulin deacetylases and αTAT1 acetyltransferase also modify other proteins [34,35], possibly also proteins affecting microtubule dynamics. For example, HDAC6-dependent deacetylation of both α-tubulin and cortactin leads to cilia disassembly [36].

In *C. elegans* Mec-17 is highly expressed in the touch receptor neurons [37], whose neurites contain unusual large diameter microtubules composed of 15 protofilaments [38]. Both the number of assembled microtubules and the number of protofilaments in these microtubules depend upon the Mec-17 acetyltransferase [39,40,41]. The introduction of the enzymatically inactive Mec-17 (D144N) rescues microtubule instability but not alterations in protofilament number in mec-17 mutant [39,41]. Thus, the presence but not the activity of Mec-17 is sufficient to stabilize these microtubules.

Mec-17 acetyltransferase-deficient touch receptor neurons assemble heterogeneous microtubules with protofilament numbers varying between 11 to 16 [39,40]. Based on molecular dynamics simulations, it was proposed that a conserved glutamic acid at position 55 (E55) of α-tubulin could form either an intramolecular salt bridge with K40 or an intermolecular bridge with histidine 283 (H283) of an α-tubulin from the neighboring protofilament. The acetylation of K40 would lower the probability of K40–E55 interactions and favor the E55–H283 interaction, which would promote the formation of the microtubules with 15 protofilaments [40]. However, K40 α-tubulin acetylation is surely not the sole factor determining the number of protofilaments. Less than 20% of in vitro polymerized microtubules built of completely acetylated tubulin were composed of 15 protofilaments. Moreover, such 15-protofilament microtubules, although infrequent (less than 10% of microtubules) were also observed among microtubules assembled from deacetylated tubulin [17]. Additionally, acetylation did not change the microtubule structure, as was observed using cryo-electron microscopy [17]. Howes and co-authors [17] suggested that K40 acetylation may affect the interactions between microtubules and as yet unidentified MAPs (microtubule associated proteins), and thus affect microtubule architecture.

The 15-protofilament microtubules are built out of Mec-12 α-tubulin and Mec-7 β-tubulin. Mutations in either of these tubulins also affect the number of protofilaments [5,42]. Thus, not only α-tubulin with acetylatable K40 but also Mec-7 β-tubulin is important for the assembly of large microtubules in touch receptor neurons in *C. elegans*.

Early in vitro microtubule polymerization assays with tubulin purified from calf brain, and next incubated with TAT in the presence of either acetyl-CoA (acetylated tubulin) or CoA (control), suggested that K40 α-tubulin acetylation does not affect the rate of microtubule polymerization or cold-induced depolymerization [43]. However, tubulin in neurons, especially in axons, is already acetylated; thus, the control “unacetylated” tubulin used in these experiments had some level of acetylation. As it is now feasible to produce tubulin that is either nearly completely acetylated (more than 96%) or deacetylated (less than 1% acetylation), the question of whether acetylation affects the polymerization or depolymerization rates was revisited. It was shown that in vitro the acetylated tubulin self-assembles more slowly than deacetylated tubulin. Specifically, tubulin acetylation reduces the microtubule nucleation rate [44]. However, once microtubules are formed, the growth rates of the acetylated and deacetylated microtubules are similar, while the depolymerization rate is faster in the case of acetylated microtubules [44]. Portran and co-authors propose that K40 acetylation reduces lateral but not longitudinal interactions between tubulin heterodimers [44].

Moreover, under stress conditions, deacetylated microtubules break more frequently than acetylated ones, both in vitro and in vivo [32] and K40 α-tubulin acetylation reduces the number of microtubule breakages and increases microtubules flexibility by weakening the lateral interactions between neighboring protofilaments [32,44]. These authors proposed that “…by weakening interprotofilament interactions, acetylation increases lattice plasticity and limits the spread of preexisting lattice damage under repeated mechanical stress and thus protects microtubules from material fatigue or mechanical breakage.” ([32], p. 4).

Some evidence supports the view that tubulin acetylation may affect microtubules stability indirectly as well. In neurons, acetylated microtubules may be a preferred substrate of the microtubule-severing protein, katanin [45]. Similar conclusions were drawn based on katanin analysis in *Drosophila* [46]. Another microtubule severing protein, fidgetin, also seems to distinguish between stable and labile microtubules. In mouse neurons fidgetin has a preferences toward labile unacetylated microtubules as a substrate, while its ortholog in *Drosophila* acts on stable microtubules [47]. Unlike with katanin and fidgetin, the activity of the microtubule-severing protein, spastin, was not affected by the level of tubulin acetylation [45,48].

**Figure 2:**
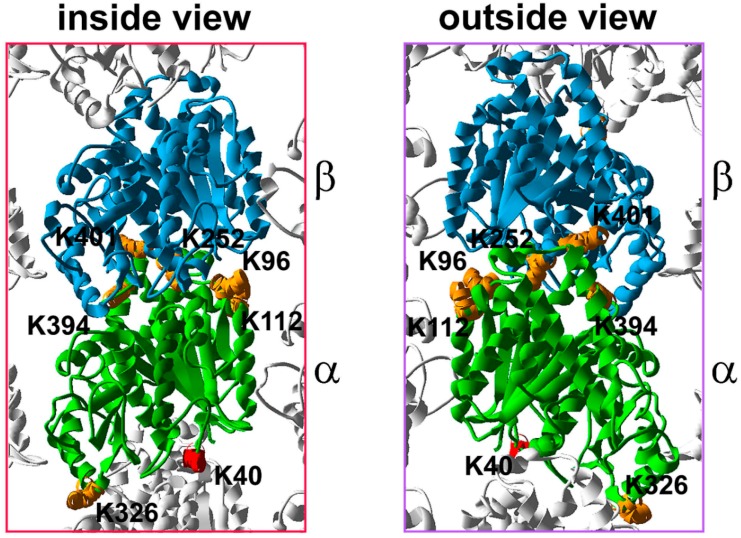
The distribution of the acetylated residues within the α,β-tubulin heterodimer. 3D model of a tubulin heterodimer within the microtubule lattice based on 3J6F.pdb (NCBI database), an inside and outside view. Putative acetylation sites are marked in orange. An approximate location of K40 is marked in red (K40 was not included in the original 3D model). α-Tubulin K96 is located within the S3 strand and builds the plus end surface, responsible for the interactions with β-tubulin of the same heterodimer. α-Tubulin K112 is located in close vicinity to the H3 surface while K326 is located within the H10 helix, and builds the minus end surface responsible for longitudinal interactions between heterodimers within the same protofilament or with γ-tubulin at the nucleation site. α-Tubulin K394 and K401 are located within the H11 helix and H11–H12 loop, respectively which are exposed on the outside surface involved in the interactions with non-tubulin partners of tubulin. K252 of β-tubulin is located within the H8 helix and builds the minus end surface.

Lysine 252 residue (K252) of β-tubulin is another acetylation site (Figure 2). Contrary to K40 of α-tubulin, acetylation of K252 occurs on free tubulin, and this reaction is catalyzed by San acetyltransferase [49]. In HeLa cells, siRNA-directed knockdown of San did not change the cold-induced microtubule depolymerization rate, but microtubules regrowth was faster in cells with a reduced level of San compared to the control cells. Accordingly, the incorporation rate of the tubulin heterodimers into microtubules during microtubules regrowth was reduced in cells expressing β-tubulin with the acetylation-mimicking mutation of K252 (K252Q) [49]. Thus, it appears that acetylation of K252 of β-tubulin regulates microtubule dynamics by reducing the rate of microtubule assembly. K252 is positioned within the H8 helix, a part of the β-tubulin minus end surface [8,50] that is involved in the interactions between two tubulins of a heterodimer. Thus, it is possible that the positively charged K252 stabilizes the tubulin heterodimer because of its neutralizing effect on the negatively charged GTP bound to α-tubulin. The K252 acetylation changes the K252 charge, and may weaken this interaction [49]. Thus, K252 acetylation may affect heterodimer formation and/or stability, and cause changes in the heterodimer conformation, and thus disfavor polymerization [49]. Further analysis, including in vitro polymerization assays, will be needed to fully evaluate the significance of the acetylation of K252 of β-tubulin.

Potential additional acetylation sites were identified on both α- and β-tubulin in a large-scale mass spectrometry analysis of the acetylome in human MV4-11 cells [51], tubulin purified from HeLa cells [52], and by a comparative analysis of brain tubulin purified from wild-type and HDAC6 knockout mice (this last approach limited the identified putative acetylation sites to lysine residues specifically deacetylated by HDAC6) [53]. The significance of the majority of the newly identified putative acetylation sites was not experimentally addressed. Site-directed mutagenesis of several α-tubulin lysine residues (K96, K112, K326, K394) identified as putative acetylation sites, suggested that acetylation of K394 may affect microtubule polymerization. When overexpressed in HeLa cells, GFP-tagged α-tubulin with either a K394Q mutation mimicking permanent acetylation, or a K394R mutation blocking the modification, was poorly incorporated into polymerizing microtubules [52]. Interestingly, expression of the GFP-α-tubulin with mutations of K326 or a double K96/K112 mutation, mimicking either permanent acetylation (K- > Q) or lack of the modification (K- > R), had the same impact on the cold-induced microtubule depolymerization rate. However, the re-growth of the completely depolymerized microtubules was delayed in cells expressing GFP-α-tubulin carrying a K96Q K112Q double mutation, or slightly delayed in cells expressing GFP-α-tubulin with either the K40R or K326Q mutations [52]. However, further analyses are needed to confirm this observation.

## 4. Methylation, Modification of the Minus End of a Subset of Mitotic Microtubules

In mitotic cells, α-tubulin of the central spindle, but not astral microtubules, is modified by trimethylation at the K40 by SET-domain-containing 2 (SETD2) methyltransferase [54]. At metaphase the trimethylated α-tubulin is enriched near the microtubule minus ends, especially near the spindle poles, and during cytokinesis in the distal ends of the midbody microtubules.

In Setd2^flox/flox^; ER-Cre MEFs, a loss of SETD2 caused mitotic aberrations, including the assembly of multipolar spindles at prometaphase, or lagging chromosomes during the separation of the genetic material at anaphase. In vitro recombinant SETD2 modifies both free tubulin heterodimers and microtubules. Thus, directly or indirectly (by regulation of the interactions with MAPs), α-tubulin K40 methylation could influence the dynamics of the microtubule minus ends. Moreover, acetylation and trimethylation could compete over the same modification site [54].

## 5. Tubulin Phosphorylation

**Figure 3:**
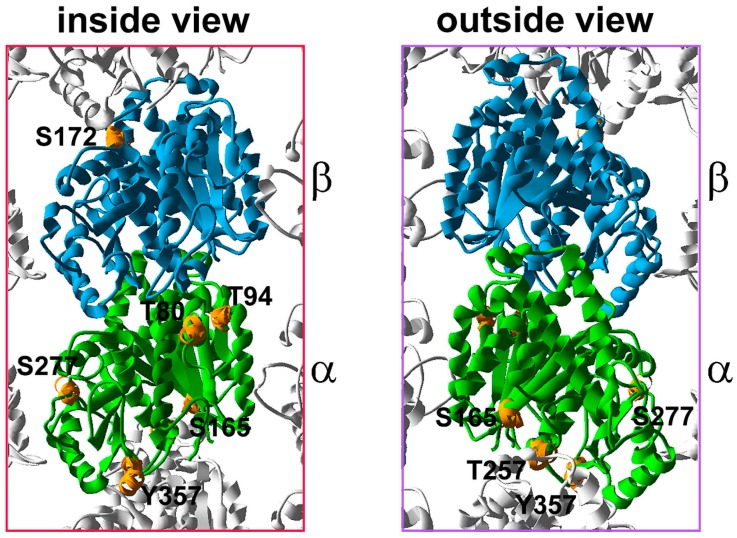
The distribution of the phosphorylated residues within the α,β-tubulin heterodimer. The 3D model of the tubulin heterodimer within the microtubule lattice is based on 3J6F.pdb (NCBI database), an inside and outside view. Putative and confirmed sites of tubulin phosphorylation are marked in orange. Luminal T80 and T94 of α-tubulin are located within the H2 helix and S3 strand, respectively. α-Tubulin S165 is located within the H4–S5 loop, near the H3 surface, but is not a part of this surface. α-Tubulin T257 is located within the H8 helix, building the minus end surface. α-Tubulin S277 is located in the S7–H9 loop, also called the M loop but according to the model proposed by Inclán and Nogales [9] S277 is not a part of the ML surface. A modification of S277 could influence the structure of the ML surface and thus affect the interactions with the neighboring protofilament. α-Tubulin Y357 is located within the S9–S10 loop. S172 of β-tubulin is located within the S5–H5 loop, called also T5 loop.

Numerous studies have shown that both α- and β-tubulin can be phosphorylated on serine (S), threonine (T), and tyrosine (Y) residues [55,56,57,58,59,60,61]. However, the physiological significance of tubulin phosphorylation has only rarely been elucidated. Recently, phosphorylated serine and threonine residues on both α- and β-tubulin were identified in a large-scale mass spectrometry analysis of tubulin purified from HeLa cells [52]. Three-dimensional modeling revealed that three of the newly identified phosphorylation sites on α-tubulin, (S277), (T257) and (Y357), may contribute to the interactions between α- and β-tubulin, either of the subsequent (T257, Y357) or neighboring dimers (S277) (Figure 3), while threonine residues (T80 and T94) are exposed to the microtubule lumen [52]. If so, acetylation of K40 of α-tubulin may not be the only luminal modification. In vivo analysis using site-directed mutagenesis of α-tubulin residues (T80, T94, T257, S277 and Y357), mimicking either lack of phosphorylation (substitution with an alanine residue) or permanent phospho-group attachment (substitution with an aspartic acid residue) revealed that only the T257D mutation prevents incorporation of the α-tubulin into polymerizing microtubules [52].

The comparison of the properties of microtubules in cells expressing mutated versions of α-tubulin showed that microtubules containing α-tubulin with phospho-mimicking mutations were generally more sensitive to cold-induced depolymerization than microtubules containing α-tubulin with mutations that prevented the phosphorylation of the corresponding residue [52]. Moreover, once depolymerized, microtubules re-polymerized faster in cells expressing α-tubulin carrying an alanine substitution, while re-polymerization was delayed in cells expressing α-tubulin with a phospho-mimicking mutation [52]. Since no in vitro data are available, it remains to be determined if changes in the status of tubulin phosphorylation have direct or indirect effects on microtubule dynamics.

Phosphorylation of the highly evolutionarily conserved serine 172 residue (S172) of β-tubulin seems to regulate microtubule polymerization. In neurons, minibrain/DYRK1a kinase phosphorylates S172 and inhibits tubulin polymerization [62]. In mammalian mitotic cells, β-tubulin is phosphorylated on S172 by a cyclin-dependent kinase, Cdk1. The phosphorylated β-tubulin or β-tubulin with a mutation mimicking permanent phosphorylation at this residue is either poorly incorporated into microtubules or not incorporated, in vitro and in vivo, respectively [63]. Thus, S172 phosphorylation may regulate the dynamics of the microtubules during mitosis by limiting the amount of soluble tubulin heterodimers available for polymerization. Also in budding yeast, either S172A or S172E substitutions in β-tubulin affect microtubule dynamics [64].

S172 is positioned within the T5 loop, a region of β-tubulin that is in close proximity to the GTP/GDP binding site, and is part of the plus end surface that mediates the assembly of the heterodimers into a protofilament [8,9]. Thus, S172 phosphorylation may affect both GTP/GDP binding and/or turnover, and the interactions between tubulin heterodimers [63].

The incorporation of tubulin into microtubules may also be blocked by phosphorylation on tyrosine residues. In activated T-cells, α-tubulin that is phosphorylated on as-yet unknown tyrosine residue(s) remains in the soluble fraction [65]. Similar data were obtained in in vitro studies with α-tubulin phosphorylated at a C-terminal tyrosine residue by insulin receptor kinase [55].

On the other hand, tubulin phosphorylation may promote microtubule growth. α6Tubulin phosphorylated on serine165 (S165) by PKCα kinase [66] or carrying a S165D phosphorylation-mimicking mutation is more efficiently incorporated into growing microtubules compared with unmodified tubulin [67]. In cells treated with a diacylglycerol (DAG)-lactone, a membrane-permeable PKC activator, or expressing an S165D α6tubulin, the duration of the microtubules growth phase was considerably longer while the phases of shrinkage and pause were shorter compared to control untreated cells or cells expressing S165N α6tubulin [67]. S165 of α6tubulin is positioned within the H4-S5 loop (Figure 3) and potentially may affect the interactions between α- and β-tubulin of the subsequent heterodimers of the protofilament during microtubule assembly [66].

It is noteworthy that some kinases may affect microtubule dynamics in a catalytic activity -independent way functioning as MAPs [68,69].

## 6. Post-Translational Modifications Specific to the Tubulin Tail

Properly folded, α- and β-tubulin molecules consist of a predominant globular core and a short, unstructured C-terminal tail. This tail can be cleaved off by subtilisin. In vitro polymerized and subtilisin-treated, tail-less microtubules showed similar resistance to cold- or dilution-induced depolymerization as the intact microtubules, but were more resistant to calcium-induced depolymerization [70].

The negatively charged tubulin tails protrude above the microtubule surface and regulate the interactions between the microtubules and some microtubule-interacting proteins such as structural MAPs, microtubule-severing proteins, and motor proteins [71,72,73]. Some of these microtubule-interacting proteins affect microtubule stability.

### 6.1. Tyrosination, Detyrosination and Δ2 Modification

A tyrosine residue is the most frequent gene encoded final amino acid residue on the α-tubulin C-terminal end. This residue can be removed from α-tubulin incorporated into microtubules by an unknown carboxypeptidase (detyrosination) [74,75], leading to the formation of so-called Glu-tubulin (from the exposed glutamic acid, the penultimate residue of α-tubulin). After microtubule depolymerization, the removed tyrosine residue can be re-ligated to free tubulin heterodimers by tubulin tyrosine ligase (TTL) [76,77,78,79,80,81]. In microtubules, the detyrosinated α-tubulin can undergo further shortening by the elimination of the penultimate glutamic acid residue [82] carried out by the cytosolic carboxypeptidases [83,84,85]. Generation of the Δ2 α-tubulin is irreversible and limits the pool of the detyrosinated α-tubulin that can be restored to the gene-encoded state.

In vivo, in contrast to the tyrosinated microtubules that undergo turnover within minutes, detyrosinated microtubules persist even for hours [86], and similarly to K40 acetylation, the level of α-tubulin detyrosination increases with time. However, detyrosination is a marker of long-lived microtubules [87], not a direct cause of the microtubule stability [3,88,89].

In vitro, the level of tubulin tyrosination/detyrosination seems to have no effect on tubulin polymerization [90], or the binding of MAPs [91,92] and has a minimal effect on the binding and severing of microtubules by the microtubule-severing protein, spastin [48]. However, recently it was shown that in mammalian astrocytes and neurons, fidgetin preferentially targets the dynamic, tyrosinated microtubules (opposite to fidgetin in *Drosophila*, where stable acetylated microtubules are the preferred substrate) [47,93]. Moreover, in neurons, phosphorylated MAP1B preferentially interacts with the tyrosinated microtubules and locally modulates microtubule dynamics [94].

α-Tubulin tyrosination also affects microtubule dynamics, by regulation of the interactions between microtubules and kinesin-13. MCAK (KIF2C), a motor-like protein that induces depolymerization of microtubules ends [95], has preferential activity for tyrosinated microtubules, both in vivo and in vitro. Microtubules assembled from the purified tyrosinated tubulin or tubulin with an engineered C-terminal tail are depolymerized more efficiently by MCAK than microtubules assembled from detyrosinated tubulin [96,97]. Moreover, uniform detyrosination of microtubules by pretreatment with carboxypeptidase protects against depolymerization by recombinant MCAK [96]. Accordingly, the phenotype of the TTL-null neurons is strikingly similar to the phenotype of KIF2A kinesin-13-null neurons [96,98]. Thus, kinesin-13 depolymerases may be using microtubules ends with tyrosinated tubulin as a preferred substrate and this effect could explain the increased longevity of the detyrosinated microtubules.

The presence of tyrosinated tubulin within microtubules also favors interactions between the microtubule plus end and microtubule plus end tracking proteins (+TIPs) that have a conserved CAP-Gly domain; CLIP170, CLIP115 and p150glued bind more strongly to tyrosinated than detyrosinated microtubules [99,100,101,102].

### 6.2. Glutamylation and Glycylation, Polymodifications of the Tubulin Tail

Glycylation and glutamylation are polymeric modifications (polymodifications) that consist of one or more glycyl (G) or glutamyl (E) residues, respectively, ligated to the glutamic acid residues within the C-terminal tail of both α- and β-tubulin. In consequence, these post-translational modifications lead to the assembly of either polyG or polyE side chains of different length [103,104]. Both α- and β-tubulin tails have several potential polymodification sites [105,106].

Tubulin polymodifications are catalyzed by enzymes related to tubulin tyrosine ligase (TTL), called tubulin tyrosine ligase-like (TTLL) [107,108,109,110]. Mammalian TTLL3, TTLL8, and TTLL10 enzymes function as mono- and polyglycylases, respectively [109,110,111] while TTLL1, 4, 5, 6, 7, 9, 11, and 13 possess glutamylase activity [107,108,112,113,114]. Some of the TTLLs initiate the glutamyl (TTLL1, TTLL4, TTLL7) or glycyl (TTLL3, TTLL8) side chain by ligation of the first side residue to the γ-carboxyl group of glutamic acid within the tubulin tail via an isopeptide bond. The initiated side-chain can be elongated leading to the formation of long glycyl (TTLL10) or glutamyl (TTLL6) side chains [107,108,109,113,114,115,116].

Tubulin glutamylation [117] and most probably also glycylation [118] are reversible modifications. The glutamyl side chains can be either shortened or removed by tubulin deglutamylases belonging to the family of cytoplasmic carboxypeptidases (CCP) [83,84,85,119,120]. In mammals, there are 6 CCP deglutamylases. Interestingly, the same enzymes can also remove the C-terminal glutamic acid residues from detyrosinated α-tubulin, creating Δ2 tubulin or even Δ3 tubulin, depending upon the number of C-terminal glutamic acid residues exposed in the detyrosinated tubulin tail [83,84,85]. Until now the identity of the tubulin deglycylase(s) remained unknown. For more detailed description of the properties of tubulin glycylases, glutamylases, and deglutamylases, please refer to recent reviews on the subject [71,72,73,121,122].

Several studies have shown that changes in the extent of tubulin polymodifications can affect the stability of microtubular structures. The ciliary axoneme is built out of nine outer doublet microtubules composed of A- and B-tubule and in motile cilia, additionally, of a pair of central microtubules (9 + 2). The site-directed mutagenesis of the potential polymodification sites within the β-tubulin C-terminal tail provided the first evidence that the levels of the tail-specific tubulin post-translational modifications may affect axoneme ultrastructure [105,123,124]. *Tetrahymena* mutants with potential modifiable glutamic acid residues replaced by the non-modifiable aspartic acids within the β-tubulin tail (β-DDDE440) assemble short cilia that lack central pair microtubules, and some B-tubules [123,124]. In zebrafish, morpholino-driven knockdown of ttll6 glutamylase causes loss of the olfactory cilia, and partial loss of the B-tubules in the pronephric cilia [125,126]. The destabilization of the B-tubule becomes more pronounced in double morphants of ttll6 and ttll3 glycylase [126]. In mice, TTLL8-dependent glycylation plays a role in the maintenance of cilia in multiciliated ependymal cells [127], while lack or dysfunction of the TTLL5 glutamylase causes production of sperm cells with an axoneme missing a single doublet number 4 [128]. In *Tetrahymena,* the overexpression of a potent β-tubulin glutamylase elongase Ttll6A leads to the assembly of short cilia with ultrastructural defects, and hyper-stabilization of the cortical and cytoplasmic microtubules [107,116]. Similarly, an increase in the level of tubulin glutamylation progressively destabilizes B-tubules in sensory cilia in *C. elegans* [129].

Since the chemical properties of glycyl and glutamyl side chains are different, it is very likely that the roles of both polymodifications are not the same. Recent evidence suggests that tubulin polymodifications influence microtubule dynamics, not directly but indirectly by the regulation of the affinity of the microtubule-interacting proteins. The in vitro analysis suggests that glutamylation may modulate the binding of the neuronal MAPs [130,131]. Moreover, both in vitro and in vivo data have shown that tubulin glutamylation regulates the extent of the microtubule fragmentation by the microtubule-severing protein, spastin.

The microtubule-severing enzymes, spastin, katanin and fidgetin, are present in most eukaryotes. In the presence of ATP, microtubule-severing proteins form a ring-like hexamer and generate internal breaks in the microtubule lattice, leading to microtubule fragmentation [132]. Depending on the presence of other proteins, short microtubular fragments: (i) can be transported; (ii) serve as templates to polymerize new microtubules and increase their number, or (iii) undergo depolymerization, leading to an increase in the pool of free tubulin.

Based on the structural analysis of spastin, it was proposed that the central pore of the hexamer interacts with the negatively charged C-terminal tail of tubulin, causing local changes in the microtubule lattice that in turn, lead to microtubule breakage [132,133]. Tubulin glutamylation increases the affinity of spastin for microtubules, and affects its enzymatic activity. The severing activity of spastin increases with an increase in the number of post-translationally added glutamic acid residues. The highest activity is observed on microtubules with 8–9 glutamates per tubulin, while longer glutamyl side-chains reduce spastin severing activity [48,134].

Some MAPs can protect microtubules against severing, for example, MAP4 and tau in neurons [135,136] and SPR2 in *Arabidopsis* [137].

## 7. Other Modifications

Some recently identified and thus unexplored tubulin post-translational modifications can also influence microtubule dynamics. Tubulin succination, the generation of the S-(2-succino) cysteine by the reaction between fumarate, the intermediate of the Krebs cycle and thiol groups of the cysteine residues [138] of α- and β-tubulin, was identified in mouse adipocytes. Mass spectrometry analysis of the in vitro modified porcine brain tubulin showed that numerous cysteine residues of both α- and β-tubulin can be modified, and that tubulin succination reduces the microtubule polymerization rate [139]. However, endogenous succinated tubulin seems to be more abundant in more dynamic microtubules [139].

Both α- and β-tubulin can also be modified by the binding of *O*-Glc-NAc (*O*-Glc-NAcylation) to serine or threonine residues [52,140]. A single *O*-Glc-NAcylated α-tubulin peptide (173–185) with two putative modification sites, serine (S178) and threonine (T179) and two partly overlapping β-tubulin peptides (216–238) encompassing several serine and threonine residues, were identified during mass spectrometry analysis of mammalian tubulin [140]. Both S178 and T179 of α-tubulin are positioned within the T5 loop that is in close proximity to the GTP binding site and is a part of the plus end surface [8,9]. Modifications of these residues could affect GTP binding and α- and β-tubulin heterodimerization [140]. Accordingly, in vitro *O*-Glc-NAcylation of α-tubulin, but not β-tubulin reduced interactions between α- and β-tubulin and dimer formation. Moreover, in vitro, both *O*-Glc-NAcylated tubulins were not incorporated into polymerized microtubules [140].

Over time, free tubulin heterodimers and newly polymerized microtubules accumulate numerous post-translational modifications that diversify their properties, including microtubule dynamics. It is well documented that the pattern of tubulin modifications changes during the progress of the cell cycle, during cell differentiation and in pathogenic processes including tumorigenesis and neurodegeneration [141,142]. Moreover, the pattern of tubulin modifications can be different in different cell types or even cell compartments or along the same microtubule. Some modifications are present only on long-lived, stable microtubules (such as α-tubulin detyrosination or acetylation), others are a hallmark of dynamic microtubules (like tyrosinated α-tubulin). Some modifications can be detected only on certain types of microtubules (methylation on spindle but not astral microtubules) or in certain cell types (glycylation in ciliated cells). The level of tubulin modifications can be regulated on multiple levels, including the expression of the modifying enzymes, regulation of the localization (adaptor proteins), the activity of the modifying enzymes and balance between modifying and de-modifying enzymes. Not surprisingly, we are only beginning to uncover the complexity of the tubulin code formed on the microtubule surface and its impact on the processes in different cell types.
